# Delayed diagnosis of lung cancer after missed vertebral metastasis on CT

**DOI:** 10.1259/bjrcr.20140001

**Published:** 2015-03-18

**Authors:** M P H Hawkesford, S Kalogrianitis

**Affiliations:** Department of Trauma and Orthopaedics, University Hospitals Birmingham NHS Foundation Trust, Queen Elizabeth Hospital Birmingham, Birmingham, UK

## Abstract

A 71-year-old man presented with a 4-month history of severe atraumatic monolateral hip pain. Radiographs were normal, and MRI had to be aborted owing to heating up of a remnant of an old spinal cord stimulator. CT revealed squamous cell lung carcinoma with widespread metastases of the spine and pelvis, causing L1 nerve root compression. In retrospect, a lytic lesion consistent with spinal metastasis was found on CT taken 5 months previously, soon after the onset of hip pain, but this was missed by the reporting radiologist at that time. This case highlights that errors in radiology reporting are inevitable, but can be minimized by using a systematic approach to carefully review all available images to avoid missing unexpected pathology.

## CLINICAL PRESENTATION

A 71-year-old man presented to the accident and emergency department of a major trauma centre with a 4-month history of worsening severe left hip pain. The pain was insidious in onset and constant, but with frequent spasmodic exacerbations even at rest. The pain had been keeping him awake at night and had become significantly worse over the past few hours. The patient described the pain as being “stabbing” or “burning” in nature, “like a red hot poker”. It was localized to the hip, with no radiation to the back or down the lower limb. The patient had been prescribed increasing strengths of opioid analgesics over the past few months, but these were no longer effective. His pain was now “10/10” in severity, despite his usual medication. The patient’s appetite was reduced, but he had not noticed any significant weight loss. He was otherwise systemically well and apyrexial. Oxygen saturations were recorded as 100% on room air, although he was tachypnoeic. On examination, there was tenderness over the left groin, but no hernia. There was no abdominal tenderness, guarding or masses, or spinal tenderness, and his breath sounds were unremarkable. He had full active range of movement, normal power and sensation of both lower limbs, with palpable pedal pulses. There was only a mild increase in pain on internal and external rotation of the left hip.

The patient had a complex history of chronic pain after an accident at work approximately 30 years ago, when he sustained a traction injury to the upper cords of the brachial plexus. This caused severe pain and paraesthesia over the right side of the neck and right upper limb. After multiple analgesic injections failed to improve these symptoms, the patient underwent implantation of a spinal cord stimulator in the right flank with wires up to the cervical spinal cord. Unfortunately, the stimulator malfunctioned and became ineffective following a road traffic collision 5 years after the implantation. The patient suffered residual right groin pain owing to battery leakage, following which the stimulator was removed. A small fragment of wire remained in the patient’s back, and this had previously been thought to be the source of the ongoing pain. Removal of this fragment had been planned 1 month prior to the patient’s most current admission, but was cancelled owing to lack of bed space.

Other past medical history included hypertension, chronic iron deficiency anaemia and a previous myocardial infarction. The patient lived alone in a warden-controlled flat since the death of his wife owing to metastatic lung cancer 10 years ago. He had been independently mobile with a stick, but was no longer able to bear his own weight owing to the pain. He was a retired builder and an ex-smoker of 33 pack-years.

## DIFFERENTIAL DIAGNOSIS

The patient was admitted under the trauma and orthopaedic team for analgesia, and for further investigation of the cause of his severe hip pain. The lack of any significant trauma in the patient’s history of presenting complaint, and the full range of movement on examination, meant that a hip fracture was unlikely. Although osteoarthritis would need to be excluded, pain at night and constant pain even at rest meant that it was also unlikely to be the underlying cause. In view of the patient’s complex history of chronic pain, an exacerbation of the pain in relation to the fragment of wire remaining *in situ* was initially felt to be the most likely cause. However, the descriptors of neuropathic pain, such as “burning” and “like a red hot poker”, indicated that lumbar disc herniation and radiculopathy also needed to be excluded.

## INVESTIGATIONS/IMAGING FINDINGS

**Figure 1.**
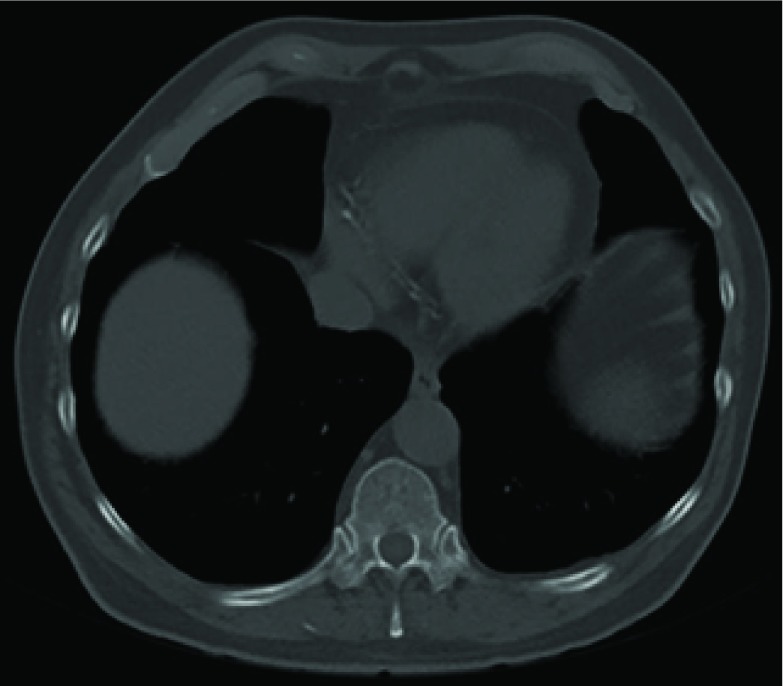
CT scan (bone window) of abdomen performed 4 months prior to admission showing metal wire fragment foreign body in the right erector spinae.

Radiographs of the pelvis and left hip showed no acute bony injury and only minor bilateral degenerative changes. CT of the abdomen had been performed 4 months prior to the admission in order to plan removal of the remaining spinal cord stimulator wire fragment, around the time of onset of the most recent left hip pain. This was reported to show a 15 × 1 mm foreign body within the right erector spinae muscles, consistent with a wire fragment (Figure 1). Basal emphysematous changes were also noted, but the report stated “no destructive bony lesions”.

MRI of the lumbar spine and pelvis was planned to exclude an L1 nerve root impingement, but the scan had to be aborted after a few minutes in the scanner as the patient complained that he could feel the metal wire heating up. The very limited image obtained from this MRI scan showed an abnormal bone marrow signal with a mild wedge compression fracture of the L1 vertebra. A staging CT of the chest, abdomen and pelvis was advised to exclude a neoplastic cause for this signal.

**Figure 2.**
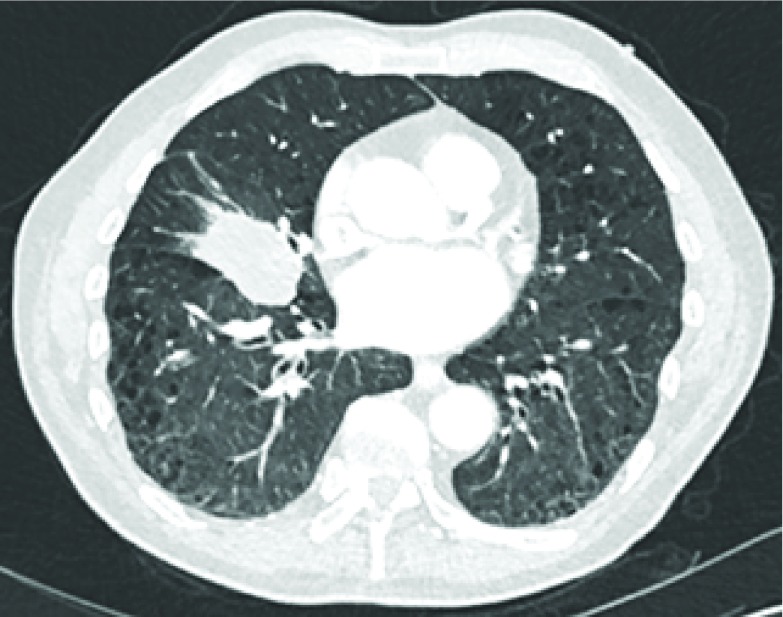
CT scan (lung window) of chest performed during admission showing a speculated soft tissue mass in the right middle lobe consistent with a primary bronchogenic carcinoma.

**Figure 3.**
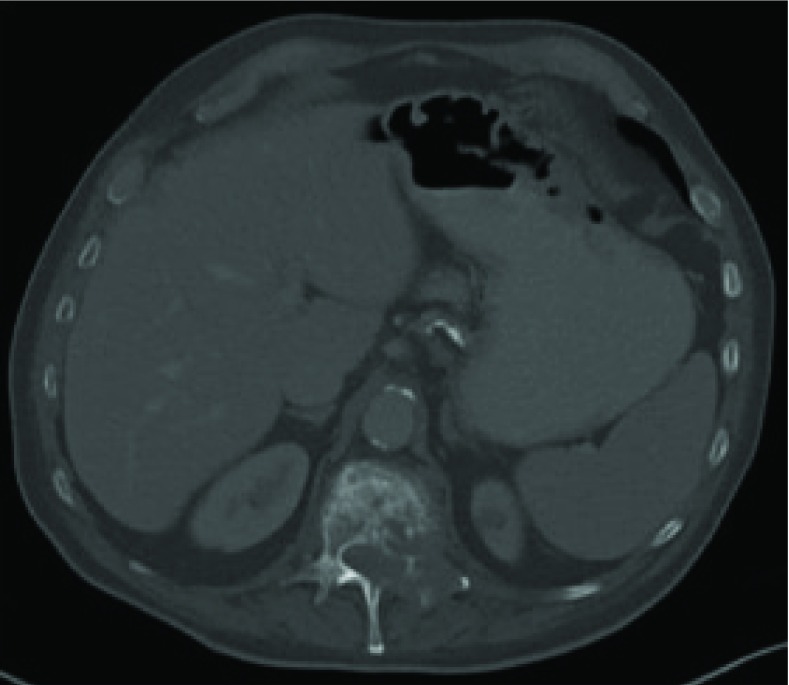
CT scan (bone window) of abdomen performed during admission showing an expansile lytic lesion of the L1 vertebra consistent with bony metastasis.

**Figure 4.**
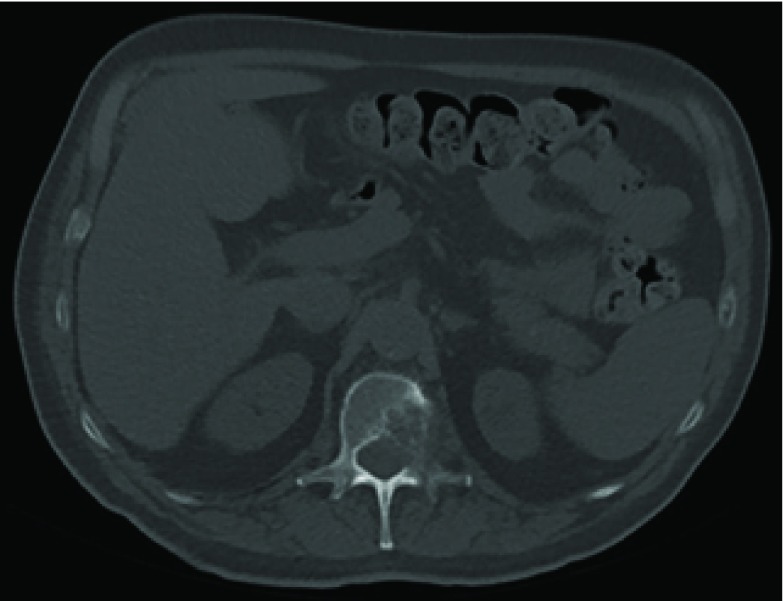
CT scan (bone window) of abdomen performed 4 months prior to admission showing a lytic lesion of the L1 vertebra consistent with bony metastasis.

The staging CT was reported to be consistent with a primary bronchogenic carcinoma in the middle lobe of the right lung, classified as T2aN1M1b (Figure 2). The L1 vertebra was noted to have collapsed, with expansile lytic lesions extending into the left pedicle and transverse process (Figure 3). The surrounding soft tissue filled the nerve root exit foramina and encroached into the bony spinal canal. There were further multiple lytic lesions in the L4 vertebral body, left pedicle of the L5 vertebra, left sacral alum and right periacetabular region. On retrospective review of the CT performed 5 months ago, a lytic lesion was seen in the left posterior aspect of the L1 vertebra, extending into the spinal canal (Figure 4).

## TREATMENT

The patient was informed of the diagnosis and he was cared for by a multidisciplinary team, including oncology, respiratory, neurosurgical and palliative care teams. His pain was initially poorly controlled despite taking high doses of opioid analgesics, and this prevented him from undergoing bronchoscopy to obtain a tissue diagnosis. He was given a single dose of palliative radiotherapy to the lumbar spine, followed by an epidural steroid, but these failed to provide any relief. The patient did improve after undergoing minimally invasive spinal fixation of the T12–L2 vertebrae and was able to start mobilizing independently. He was then able to tolerate endobronchial biopsy, which confirmed squamous cell carcinoma. Outpatient chemotherapy was planned and arrangements were initiated for the patient’s discharge from the hospital.

## OUTCOME AND FOLLOW-UP

While the discharge plans were being made, the patient developed hospital-acquired pneumonia. Despite treatment with intravenous antibiotics, his clinical condition deteriorated rapidly and he died 2 months after his admission. The cause of death was listed as bronchopneumonia leading to congestive cardiac failure, with metastatic squamous cell carcinoma of the lung, previous myocardial infarct and hypertension, all contributing to his death.

## Discussion

The patient was diagnosed with metastatic lung cancer after he was found to have metastatic lesions in his spine on CT. He had undergone an abdominal CT 5 months ago and this first scan was originally reported by a consultant radiologist to show “no destructive lesions”. On review of the original CT images, the destructive bony metastasis is clearly seen (Figure 4). While perfect accuracy should be the gold standard in reporting of radiological imaging, human error is unavoidable,^1^ and errors, discrepancies and ambiguities do occur in reporting of radiological images.^2^ A review article in 2007 found that reporting error rates among radiologists were between 3% and 5% on average.^3^ In a study of radiology registrars reporting CTs in a major trauma centre in South Africa, scans of the abdomen and pelvis elicited an error rate of 33%, the highest compared with other body regions such as the head and spine.^4^

There are a number of generic factors that can have a negative impact on the accuracy of radiology reporting, and these can be divided into causes of error attributed to the individual (the radiologist) and those attributed to the system.^1,5^ Of these causative factors, “under-reading” (*i.e.* the finding was identifiable, but was missed) by the reporting radiologist is evident. Perceptual errors constitute the bulk of radiology reporting errors,^5^ and of these, false-negative readings (missed findings) are the most common errors seen.^4^ One particular type of perceptual error is “satisfaction of search”. This is when the detection of one abnormality on a radiographic study results in premature termination of the search, allowing for the possibility of missing other related or unrelated abnormalities.^5,6^ Inadequacy of clinical information has been shown to be a significant systemic cause of reporting error, and in one study, knowledge of pertinent clinical history significantly increased the accuracy of chest radiograph interpretations by consultant radiologists from 38% to 84%.^7^ The clinical history given on our patient’s initial CT request stated, “TEN[S] machine placed and removed 14 years [ago] in December 13... part of remaining machine removed. Any pieces still [*in situ*]?” No history of back pain or any clinical suspicion of a bony lesion in the spine was given, and this is likely to have contributed to the satisfaction of search. The radiologist was asked to focus on a specific clinical question, which he/she answered.

A further systemic cause that may be pertinent in this case is excess workload, which has been shown to increase the likelihood of errors in radiological reporting.^4,6^ One particular example highlighting this is of a consultant radiologist in the USA, who was sued for missing a case of breast cancer on a mammogram.^8^ It was felt that the radiologist had read too many radiographs on that day, showing “a wanton disregard of patient well-being by sacrificing quality of patient care for volume in order to maximize revenue”. A study in 2014 also found that disruptions, such as a high volume of telephone calls, increased the risk of diagnostic error.^9^ The patient underwent both his CT scans at a major trauma centre, a busy teaching hospital. It is reasonable to assume that the reporting radiologist would have had a high workload along with a high number of phone calls and other distractions, and these probably could have contributed to this radiological reporting error.

The overall prognosis for all types of lung cancer is poor, with 1-, 5- and 10-year survival rates of 32%, 10% and 5%, respectively.^10^ For non-small-cell lung cancer in particular, 5-year survival rates vary widely depending on the stage of cancer, from 58% to 73% for Stage 1A (very early lung cancer), and between 2% and 13% for Stage 4 (metastatic lung cancer).^10^ The bony metastases in the spine that were seen retrospectively on the initial CT indicate that this patient already had Stage 4 lung cancer, and therefore the subsequent 5-month delay in diagnosis would not have had any negative effect on his poor long-term prognosis or options for treatment. An earlier diagnosis may, however, have given the patient peace of mind as to the cause of his deteriorating chronic pain and allowed for earlier management of the pain. It would also have given him longer to come to terms with his incurable diagnosis and more time to put his affairs in order.

## LEARNING POINTS

Errors in radiology reporting are unavoidable, but there are a number of contributing causative factors. In this case, several factors may have played a role in the erroneous reporting of the patient’s initial CT, including the high workload and busy environment of a major trauma centre, the relative complexity of abdominal CT imaging interpretation, and the limited clinical history and focused clinical question, leading to “satisfaction of search”. This led to a 5-month delay in the diagnosis of metastatic lung cancer, which significantly reduced the length of time the patient had to come to terms with his diagnosis and put his affairs in order. When assessing radiological imaging, it is a necessity to evaluate all the images available in a study using a systematic approach and consider that a patient may have unexpected pathology in addition to that which is most obvious.
